# Parent escalation of care for the deteriorating child in hospital: A health‐care improvement study

**DOI:** 10.1111/hex.12938

**Published:** 2019-07-16

**Authors:** Fenella J. Gill, Gavin D. Leslie, Andrea P. Marshall

**Affiliations:** ^1^ School of Nursing, Midwifery & Paramedicine, Faculty of Health Sciences Curtin University Perth Western Australia Australia; ^2^ Perth Children's Hospital Child & Adolescent Health Services Perth Western Australia Australia; ^3^ School of Nursing and Midwifery Clinical Chair Gold Coast Health Southport Queensland Australia; ^4^ School of Nursing and Midwifery and Menzies Health Institute Queensland Centre for Health Practice Innovation Southport Queensland Australia; ^5^ Nursing and Midwifery Education and Research Unit, Gold Coast Hospital and Health Service Gold Coast University Hospital Southport Queensland Australia

**Keywords:** clinical deterioration, evaluation, family involvement, implementation, paediatric, parent concern

## Abstract

**Objective:**

To evaluate the implementation of an intervention for parents to escalate care if concerned about their child's clinical condition.

**Design:**

Mixed‐methods health‐care improvement approach guided by the Theoretical Domains Framework.

**Methods:**

Implementation of the ‘Calling for Help’ (C4H) intervention was informed by previously identified barriers and facilitators. Evaluation involved audit, review of clinical deterioration incidents, interviews and focus groups.

**Setting:**

Australian specialist paediatric hospital.

**Participants:**

Convenience sample of 75 parents from inpatient areas during the audit, interviews with ten parents who had expressed concern about their child's clinical condition; five focus groups with 35 ward nurses.

**Main outcome measures:**

Parent awareness and utilization of C4H, parent and nurse views of factors influencing implementation.

**Results:**

Parent awareness of C4H improved to 35% (25/75). Parent concern was documented prior to 21/174 (12%) clinical deterioration events. All interviewed parents and nurses who participated in focus groups were positive about C4H. Parents preferred to be informed about C4H by nurses, but nurses described this as time‐consuming and selectively chose parents who they believed would benefit most. Parents and nurses described frustrations with and trepidation in escalating care. Nurses had used C4H to expedite urgent medical review.

**Conclusions:**

There was an improvement in the level of parent awareness of C4H, which was viewed positively by parents and nurses alike. To achieve a high level of parent awareness in a sustainable way, a multifaceted approach is required. Further strategies will be required for parents to feel confident enough to use C4H and to address interprofessional communication barriers.

## 1 INTRODUCTION

Internationally, various models of care are used for involving families in the early recognition and response to clinical deterioration of hospitalized patients. The intent for these models is to enable family to raise concerns about their relative's deteriorating clinical condition in a way that overcomes hospital hierarchical structures and the inequitable relationships that exist between health professionals and families. A number of programmes have been developed to assist health services implement a process to enable family escalation of care.^1^, ^2^, ^3^ Despite these initiatives being widely publicized, there remain few reports about their implementation or evaluation. Three reviews^4^, ^5^, ^6^ found 14 reports describing implementation of family escalation of care processes in the United States of America (US) and United Kingdom (UK) contexts. Key findings were that family feedback was uniformly positive, and in particular, families were happy that there was a process in place for them to voice concerns irrespective of whether or not they initiated escalation of care.^5^ On the other hand, staff views about patient or family escalation of care have been mixed, with the main concerns being about the potential for inappropriate or excessive family escalation of care calls placing a strain on already limited and under‐resourced rapid response systems.^7^

Family escalation of care process evaluation measures includes percentage of patients, carers and family members who are aware of the escalation process and the rate of calls per 1000 patient separations.^8^ These metrics can be hard to interpret; for example, few or no calls may actually reflect inadequate implementation, and conversely, a high number of calls may indicate either successful implementation or a hospital organizational failure. Brady et al^9^ reported a US children's hospital six‐year experience of family‐activated escalation of care. Their findings reinforced that utilization of the family‐activated process was uncommon, yet patient clinical deterioration may have been missed without the escalation steps taken by families. Communication failures such as lack of response by health‐care professionals and dismissive interactions between the clinical team and families were described. Another US evaluation echoed these findings and added that family initiated escalation of care occurred when patients were less severely unwell compared with clinician escalation of care.^10^ The involvement of families in recognition of and response to clinical deterioration appears to promote more timely escalation of care, yet a growing number of reports about communication concerns^11^, ^12^ emphasize the need for deeper examination beyond the number and nature of calls by families.^13^

The involvement of families in safety, especially in escalating care for clinical deterioration, is recognized as complex and difficult to achieve in practice.^14^, ^15^ The ‘complexity’ itself relates to the several interacting components within the intervention and also the interaction of the intervention with the health‐care context.^16^ Health‐care improvement is a term used to describe a systematic approach to increase the quality, safety and value of health‐care services.^17^ Health‐care improvement approaches require a theoretical underpinning, typically use mixed‐methods designs and include identification of factors that impact intervention uptake.^17^ Also key is for researchers to work closely with stakeholders and knowledge users.^18^

The PARTNER Project was initiated opportunistically to systematically examine and address the pragmatic implementation of a process for parent involvement in escalating care for the deteriorating child (Calling for Help—C4H) in an Australian paediatric hospital. A four‐part process described by French et al^19^ for developing complex interventions was used. Previously reported were parts (a) *Who needs to do what differently?* and (b) *Which barriers and facilitators needed to be addressed?* 14 The work revealed a low level of parent awareness of the C4H process and human behavioural and system‐based barriers and facilitators.^14^ Reported in the current article are parts (c) *Which intervention components could overcome the modifiable barriers and enhance the facilitators?* and (d) *How can behaviour change be measured and understood?*

## 2 METHODS

The health‐care improvement principles used in the PARTNER Project included a Study Steering Group of researchers, stakeholders and knowledge users to inform and guide the mixed‐methods study. The use of the Theoretical Domains Framework^19^, ^20^ provided a framework to first theoretically investigate barriers and facilitators and systematically inform the implementation strategy components and the evaluation. The SQUIRE 2.0 reporting guidelines^17^ were followed. Both Health Service (2015136EP) and University (HR191/2015) Human Research Ethics Committees approved the study protocol.

### 2.1 Context

The study site was a 200‐bed specialist paediatric hospital serving a population of half a million children and young people in Western Australia. The Rapid Response System operated as a two‐tiered system, with criteria for activation consistent with national recommendations and policy.^21^, ^22^ The Children's Early Warning Tool^23^ was introduced in 2011 as a track and trigger chart for measuring and documenting vital signs. The total score is a trigger for escalation of care as the score increases (maximum score 20—threshold score of 8 for review by a Medical Emergency Team [MET]). There were separate instructions to place an emergency call for urgent clinical concern, but no explicit provision for responding to parent concern.

### 2.2 Intervention

**Figure 1:**
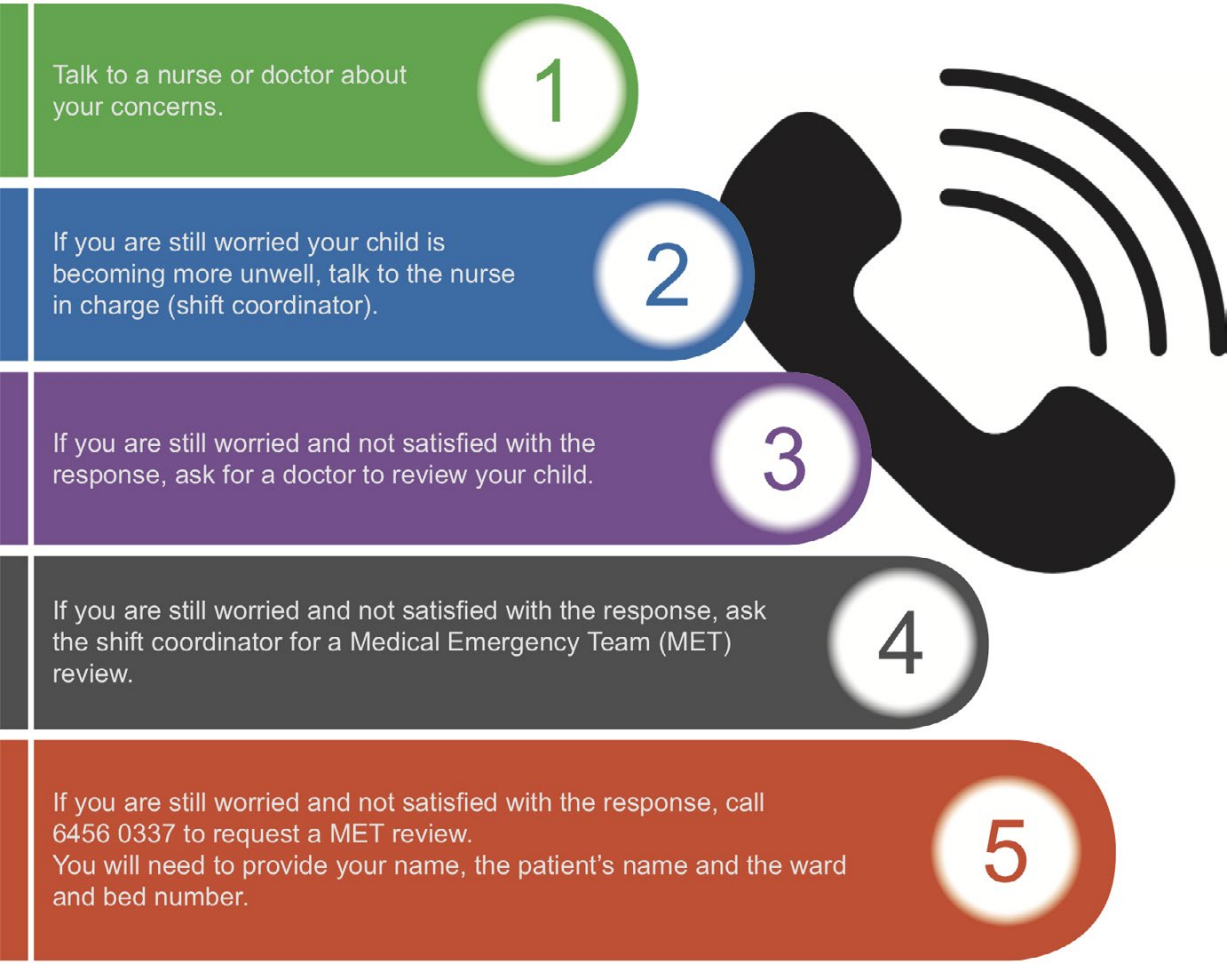
Calling for Help

The separate parent escalation of care process, C4H, was initially developed in consultation with the health service Consumer Advisory Council and involved five steps for parents to incrementally escalate their concern about clinical deterioration (Figure 1). Following updates to the existing Rapid Response System, policies and clinical guidelines and printing of parent information brochures, C4H was introduced into practice in 2015 prior to this study being undertaken.

The initial evaluation and identification of barriers and facilitators to implementation (Parts 1 and 2) enabled the key issues related to the introduction of C4H to be classified and mapped to nine domains of the Theoretical Domains Framework: knowledge; skills; social/professional role and identity; beliefs about capabilities; beliefs about consequences; memory, attention and decision processes; environmental context and resources; motivation and goals; and behavioural regulation. Using this theoretical approach supported systematic identification of key human and system factors that had influenced the introduction of C4H. A multifaceted implementation strategy was then developed to address the key issues, recognizing that to change behaviour the selected strategy should draw on theories of behaviour and behaviour change.^24^ Implementation strategies are more likely to be effective if they target causal determinants. The Michie et al matrix 24 and Cane et al hierarchy of behaviour change techniques^25^ guided the researchers to select implementation strategies that were appropriate to address the issues and also relevant and appropriate for the study context.

The researchers presented the findings and suggested a multifaceted implementation strategy to the Study Steering Group. The proposed strategy was agreed upon, and a decision was made to proceed. Table 1 shows a model to explain the implementation strategy, the factors each component addressed, the intended mechanisms of action and the components delivered, separated into those that were known to be delivered as intended (by the researchers) and those delivered by others where the dose or fidelity was not known. From 1 June 2016, implementation of the C4H intervention took place across the inpatient areas of the hospital.

**Table 1:** C4H implementation model

Implementation strategy	Barriers (B) and facilitators (F) addressed	Intended mechanisms of action	Components delivered as intended	Components with unknown fidelity or dose
Communications about changes to intervention and implementation to nurses, doctors, allied health staff	(B) Staff were uncertain that C4H had commenced (B) Staff viewed that hospital leadership support was lacking	Multifaceted strategy to deliver information Visible demonstration of hospital leadership support	Executive Director directive C4H commenced 1 June 2016 via staff email Scheduled and opportunistic presentations by Researchers to groups and teams	Scheduled and opportunistic presentations by Educators to groups and teams including staff annual resuscitation training programme
Posters redesigned and printed in colour	(B) Parents low level of knowledge (B) Staff were uncertain that C4H had commenced (B) Nurses viewed informing parents about C4H at time of admission placed an additional burden on parents (B) Nurses recommended using multiple communication strategies including before admission to hospital	Multifaceted strategy to deliver information Environmental changes to facilitate behaviour	Posters displayed on walls at each patient bed area, ward corridors, family waiting areas, outside and in each of the hospital public lifts	Parents read and understood information on posters
Brochures redesigned and printed in DL format			Delivered brochures to each ward	Ward Clinical Nurse Managers and Staff Development Nurses instructed nurses to inform families about C4H at the time of admission and provided the brochure
C4H Information included in family handbook			Updated family handbook	Family handbook posted to families prior to booked hospital admissions
Media releases and social media marketing			Researchers arranged: Local newspaper features Radio interview about the project and C4H implementation Hospital Facebook posts	Parent and staff exposed to media
Ward Champions	(F) C4H is a good fit with staff's family‐centred care practices (F) Staff viewed C4H added to patient safety (F) C4H viewed by all to be in the best interest of the child (F) Parents were able to describe signs of deterioration B) Staff doubted parents' skills to recognize deterioration and to escalate care	Modelling and demonstration of behaviour to others Social process of encouragement pressure and persuasive communication	There was one ward where more than 50% of MET calls occurred. Key nurses working on this ward were identified as C4H Champions. Researchers were in weekly contact with Champions during June and July	Ward Champions provided extra reinforcement of key messages to nurses on their ward were positive role models and points of contact for any questions by nurses.
Staff reminders	(B) Staff were uncertain that C4H had commenced (B)Staff viewed hospital leadership support was lacking F) C4H is a good fit with staff's family‐centred care practices (F) Staff viewed C4H added to patient safety	Visible demonstration of hospital leadership support Multifaceted strategy to deliver information	Reminders to Heads of clinical departments and services, Clinical Nurse Managers, Staff Development Nurses and Ward Champions	Verbal and email communication to medical and nursing staff that C4H had commenced and for nurses to inform families and provide brochures
Nursing documentation prompt	(B) Nurses did not always inform families about C4H (F) C4H is a good fit with staff's family‐centred care practices (F) Staff viewed C4H added to patient safety	Environmental change to prompt behaviour	Nursing documentation changes included prompt to talk to families about C4H at time of admission to ward and transfer from PICU to wards	Nurses were prompted to talk to families about C4H
Audit parent awareness after 6 months	(B) Parents low level of knowledge	Organizational goal setting of 50% parent awareness reported by audit and feedback Staff motivation	Goal shared with staff that by 6 months 50% of parents present with their child in wards would be aware of the C4H process Audit undertaken	All staff were aware of goal
Feedback to staff about C4H use	(B) Parents, children, doctors and nurses perceived there was potential for inappropriate calls and overuse of resources (B) Staff doubts about parents' capability or skills to recognize deterioration and to escalate care (B) Staff were concerned about possible repercussions if they missed clinical deterioration (B) Parents, children, doctors and nurses thought it would be difficult for parents to speak up and bypass the traditional hospital culture	Feedback on C4H use: frequency and nature of calls including any where staff missed clinical deterioration	MET call data were collected and reviewed. Researchers provided updates to staff about any C4H use	All staff were informed of feedback
Adapted C4H process for Emergency Department short stay ward	(B) Inpatient C4H steps not appropriate for the context	Tailored for context	Adapted process agreed and posters displayed on walls by patient bedsides	Nurses informed families about C4H

### 2.3 Study of implementation

To measure the effectiveness of the revised C4H implementation, an evaluation was undertaken after six months. Combining quantitative and qualitative methods,^26^ data collection included an audit of parent awareness of C4H, review of patient health records, interviews with parents and focus groups with ward nurses (the health professional group who interacted most closely with parents in hospital and had experience in communication about C4H). The audit of parent awareness of C4H was undertaken over two days in November 2016 and involved asking all parents who accompanied their child in ward areas on the days of the audit if they were aware of the C4H process. If they were aware of C4H, parents were asked how they had been informed. Health records of patients who had received MET calls in the six months following the revised implementation of C4H were analysed to describe call characteristics and identify how parents had been involved in the escalation of care in the eight hours prior to the MET call being placed. Key user groups of C4H were purposively selected; from the patient health record review, parents were identified as potential participants for interview if it had been documented that they were concerned about their child's clinical condition prior to the MET call being placed. Parents were interviewed in person or by telephone. The other key user group was ward nurses who volunteered to participate in focus groups conducted at the hospital.

### 2.4 Data analysis

Results from the parent awareness audit and patient record review were collated using descriptive statistics. Reporting of the qualitative component followed the COREQ checklist for interviews and focus groups.^27^ The lead researcher (a female PhD qualified nurse researcher experienced with interviews and focus groups) collected data for all interviews and focus groups and whilst known to some nurse participants did not work with or influence their work. The duration of parent interviews was between 20 and 50 minutes. These were audio‐recorded and transcribed verbatim. The duration of nurse focus groups was between 40 and 55 minutes. These data were recorded as notes. Verbal checks with participants were undertaken during each focus group and interview to confirm the researchers' understanding and additional explanations provided whenever queries arose.

A semi‐structured interview guide focused the qualitative evaluation to previously identified factors that had influenced the original implementation in 2015 (see interview guide Appendix S1). Of most interest was whether these issues had been resolved or remained and whether new issues had arisen. Hseih and Shannon's directed approach to content analysis^28^ was followed. All data that represented predetermined codes were first coded. Any data that could not be coded were identified and analysed to determine whether they represented a new category or subcategory of an existing code. A second member of the research team was present during data collection and checked and confirmed coding of data. There were no coding disagreements. Data saturation occurred when no new issues were identified after three nurse focus groups. Whilst the entire available parent cohort was interviewed, data saturation was not able to be confirmed within the recruited sample. The researchers' interpretation of the findings was presented and confirmed with the Study Steering Group which included health consumer representatives and ward nurses.

## 3 RESULTS

### 3.1 Parent awareness audit

A convenience sample of 75 parents who were at the patient bedside in 11 inpatient areas was identified. Of this, 25 (35%) were aware of C4H. For approximately 50% of parents, this was their first hospital admission, suggesting they had no prior knowledge of C4H. Seventeen (68%) parents reported they had become aware of the C4H process by reading the posters on display.

### 3.2 Patient health record review

There were 174 calls placed for a MET review over the six‐month evaluation period, and each of the 109 patient health records was reviewed (33 patients received more than one MET call). Of these calls, 172 were placed by staff (usually nurses) using the staff process, one call was by a nurse using the C4H telephone number (rather than using the staff process) and one call by a parent using the C4H telephone number. Parents were present at the time of the MET call for 102 (69%) of the calls. Parent concerns prior to the MET call being placed by staff were recorded for 21 (12%) occasions. Of these, there were eight occasions when a parent had requested a MET review, or the parent concern had prompted the MET call. There were also two occasions noted where nurses had acknowledged parents' concern and advised parents to place a MET call. There was only one occasion documented when a parent had directly called for a MET review.

Following MET reviews on 153/174 (88%) occasions, the patient remained on the ward. Three patients required surgery, and 18 patients were transferred to the Paediatric Intensive Care Unit (PICU) where one patient later died. On one occasion when parent concern had prompted staff to call for a MET review, the patient was subsequently transferred to the PICU. The outcome following the one parent direct call for a MET review was that the patient received additional analgesia medication and remained on the ward.

### 3.3 Interviews and focus groups

Fifteen parents were contacted and invited to participate. Three parents declined to be interviewed, and two did not respond. Ten parents (all female) who had been concerned about their child's deteriorating clinical condition were interviewed. The parent participant ID and their children's characteristics are summarized in Table 2, which shows 8/10 patients had complex health conditions and had between one and nine MET calls placed, and on four occasions, it was the parent who prompted or requested a MET call.

**Table 2:** Parents interviewed and their children's characteristics

Parent	Patient gender	Patient age (years, months)	Reason for this admission	Medical history	Number of MET calls	MET caller
P1	M	4 y 5 m	Respiratory illness	Immunodeficiency Post‐haemopoietic transplant	9 calls during 2 admissions	Nurse
P2	F	2 y 2 m	Respiratory illness	Nil	1	Nurse
P3	F	2 y 6 m	Seizures	Complex cardiac	1	Nurse
P4	M	2 y 11 m	Burns	Nil	5	Nurse
P5	M	11 y 9 m	Seizures	Complex neurodevelopmental	2	Nurse one call Doctor one call
P6	M	7 y	Respiratory illness	Complex syndrome	1	Parent prompted Nurse
P7	M	9 m	Respiratory illness	Complex neurodevelopmental	4	Nurse
P8	F	3 y	Respiratory illness	Congenital respiratory Tracheostomy and mechanically ventilated	1	Parent requested Nurse to call
P9	F	3 y 5 m	Respiratory illness	Congenital respiratory	1	Parent prompted Nurse
P10	F	3 y 8 m	Hypertension	Renal artery stenosis	2	Parent prompted Nurse

Five focus groups were held with a total of 35 registered nurses who worked on medical, surgical, adolescent and oncology wards. The nurses' clinical experience ranged from less than 1 year to more than 40 years.

Broad categories were developed form the parent interviews and nurse focus groups and included C4H facilitators of awareness, positive experiences and aligning with family‐centred care practices. Barriers to C4H were the traditional hospital hierarchy, nurses not informing parents about C4H as intended, remaining nurse concerns about parents using C4H and nurses being selective about which parents to inform. A further category covered recommendations to promote C4H sustainability. Quotes supporting the interview and focus group findings are presented in Table 3 with parent participant ID matched to Table 2 parent and their children's characteristics.

**Table 3:** Iterviews and focus groups supporting quotes

Categories	Parents interviewed	Nurses focus groups
Facilitator—Positive about C4H experiences	‘I just mentioned to the nurse that she didn't look right and that's when the nurse said she would get a MET call’ (P3) ‘yeah it was a very good thing to have that option’ (P10)	‘The family made the call and it worked well’ ‘There were concerns at first – but it [implementation of C4H] has not had any great effect so not a problem’.
Facilitator—aligning with Family Centre Care practice	‘I was concerned ‘cause it was abnormal for her and she's continuously sat monitored when she's asleep at home… we're very familiar with what she looks like being a very medically complicated child’ (P9)	‘If you think your child is sicker let me know’ ‘It gives parents the control back’ ‘Parents can take the lead’. ‘It is not a big issue here with a good culture, I can see the value elsewhere’.
Barrier—bypassing hospital hierarchy Challenging to use C4H process Delays in escalation	For families who are less confident or experienced; ‘there is always a chain of command you've got to go through’ (P1) ‘I was confident and I was constantly involved asking the doctors this and that so it was quite easy for me just ‘cause I'm, might be a bit of a confident person with stuff like that where other people might not be’ (P4) For parents who were experienced ‘I actually didn't call the number which in hindsight I probably should have but the reason I didn't was from a professional courtesy perspective … relying on the system and having faith that the system would work”...I didn't want to put the nurses in a compromising position’ (P9)	‘It would be confronting for new parents or parents with a sick child, it may cause unnecessary anxiety’.
	‘I felt like I was fighting to get what she should have been getting in the first place’ (P4). ‘as soon as she [the nurse] alerted me to it I used it straight away because things were so bad’ (P4)	‘When the doctors aren't listening to us’.
Barrier—Nurses did not inform parents as intended	‘She showed me the steps and she showed me the number I could call, so I then called that number’ (P10)	‘I can achieve a lot of things in the time it takes to go through C4H’ ‐ ‘its not done on our [same day care] ward’. ‘I forget’ or ‘I don't think to, not in the front of my mind’ ‘I give out the Health Facts but don't talk to them about it’ ‘I go about it in a different way, I say if you think your child is sicker let me know’.
Barrier—Nurses concern that parents may call without escalating through C4H steps		‘They may not like what the nurse is planning or doing and bypass the nurse’. ‘I’m worried about the overuse of resources if parents call a MET’.
Barrier—Nurses were selective about which parents to inform		‘It depends on different situations’. ‘It would be too much for the family to take in’ ‘… so much to cover on admission’
Recommendations to promote C4H sustainability	Be informed by nurses at admission; ‘the nurses to say well you know if you don't like it or don't feel like you're getting enough answers call this number’ (P1) More nurses are needed ‘nurses are so under the pump’ (P5) A more efficient way to escalate care for complex patients ‘nothing happens because this doctor needs to call the doctor that could be called in the first place’ (P8)	‘Other health professionals can be involved – not just by nurses but doctors and allied health too’ Display posters in more public areas such as the theatre waiting area, public toilets – ‘…on the back of toilet doors’, ‘…an infographic for non‐English speaking families’ and [parents from cultures] ‘…who won't speak up or complain’

### 3.4 C4H facilitators

#### 3.4.1 Awareness

Eight parents (80%) were aware of the C4H process at the time of their stay in hospital and had become aware through a combination of the implementation components. These included the following: reading posters (five), being informed by other parents (one), being informed by nurses (three) or informed by a doctor (one). All of the nurses who participated in the focus groups were aware of the C4H process.

#### 3.4.2 Positive experiences

Parents were positive about using the C4H process when they had been concerned about their child's deteriorating clinical deterioration. Nurses were also positive and perceived that having an organization wide process for parents to be able to raise their concerns was a good thing. Some nurses acknowledged they had initially held some reservations about the introduction of C4H and had anticipated there may have been multiple calls by parents. Most nurses had been informed there had been one parent‐activated MET call since the revised implementation of C4H.

#### 3.4.3 Aligning with family‐centred care practice

There were examples of how C4H aligned with family‐centred care practices which included nurses and doctors working closely with parents and listening to parents' concerns. Parents were positive about their involvement in their child's care and relationships with health professionals caring for their child. However, several parents recounted examples of nurses being too busy which resulted in delayed responses to their concerns. Nurses identified how C4H steps 1‐3 reflected their normal interactions with parents. They explained how they encouraged parents to speak with them if they were concerned about their child's condition. Nurses acknowledged that parents knew their child best, they could detect early deterioration, and the C4H assisted in restoring parents' sense of control in hospital. They further explained that it was helpful to be able to advise parents that there was a formal process to request an urgent medical review if they remained concerned about their child's condition. However, they considered that the most benefit for the C4H process would be in other health‐care settings where parents' input to patient assessment was not usual practice and may not be so welcomed (such as in a general hospital).

### 3.5 C4H barriers

#### 3.5.1 Bypassing hospital traditional hierarchy

Both parents and nurses perceived that it would be a difficult step for a parent to actually use the C4H steps to request a MET review which involved a challenge to the traditional hospital hierarchy. Importantly, this was acknowledged as difficult both for families who were less confident or experienced in the health‐care system as well as those who were more experienced and confident such as health professionals.

#### 3.5.2 Delays

Parents had already identified how busy nurses appeared, making it difficult for parents to speak to a nurse to communicate concerns about their child. Several parents described how their experiences of delays or lack of action by both nurses and doctors in escalating care prior to a MET call being placed. Based on these recounted experiences, two parents were referred to make complaints about their experiences. Nurses also reported being frustrated by insufficient or delayed responses by doctors to their attempts at escalation of care. There were recounts by nurses using C4H as an additional way to obtain an urgent review by instructing parents to place a MET call, and there was an occasion when a nurse placed a C4H call herself. The nurse used the C4H process as an alternative way to obtain a review by the MET when she was concerned about delayed response to her request to review the patient by the treating medical team.

#### 3.5.3 Nurses not informing parents about C4H

The intended process was that during admission to a ward nurses informed parents about the C4H process (prompted by the admission checklist) and provided a brochure. It appeared that this was not always practised, with a number of reasons provided. Some nurses remained concerned that parents may use the process to seek another opinion or choose Step 5 (See Figure 1) without escalating care through the steps. Nurses explained how in order to mitigate this perceived risk they provided comprehensive explanations to parents, which was very time‐consuming. Nurses also identified that they sometimes forgot to inform parents.

Another reason for not informing parents about C4H was that nurses were selective about who they informed, deciding themselves which parents would most benefit from the information. It was considered not practical to talk to all parents at the intended time of the child's admission. This was especially relevant to short stay surgical areas where nursing workload could involve up to 50 admissions in a day. Nurses described how they prioritized information they provided. They chose to inform parents who would benefit most from the information such as if the child was sicker and parents who were worried or unhappy with their child's care. Parents who were perceived to benefit less from receiving C4H information were those who nurses considered may be overwhelmed with information and it may worry them, or if the child appeared well and so it was considered unnecessary. Other reasons for not informing parents were if the admission to the ward occurred at night and there was limited time for communication, if the parents did not understand English and if they considered the parents might use the process inappropriately.

#### 3.5.4 Recommendations to promote C4H sustainability

Parents confirmed their preferred way to be informed about C4H was for nurses to explain it at the time of admission to the ward. There was acknowledgement that nurses were extremely busy and a need to increase the number of nurses working in some of the ward areas. For children with complex care needs, some parents recommended that there should to be a more efficient system for escalating care for their children.

In exploring how to further embed the C4H process to increase parent awareness and ensure sustainability, nurses recommended informing parents in critical care areas when nurses may have more time to talk to parents before they are admitted to a ward. Parents recommended to display posters in more public areas, include brochures in laminated form at each bedside and include in admission packs. Parents also suggested increasing awareness of the C4H process by developing alternative resources to support vulnerable groups such as parents with low English proficiency (LEP), low health literacy and from cultures where questioning health care is not the norm.

## 4 DISCUSSION

This article reports the implementation and evaluation of a complex intervention; the C4H process for parent escalation of care for the deteriorating child in hospital. The Theoretical Domains Framework supported the purposeful identification of factors influencing implementation and enabled a multifaceted implementation strategy to be designed. The intended components included communications, audit, goal setting and feedback, role modelling, tailoring and reminders. The context was a paediatric hospital environment and implementation involved researchers, educators and clinical staff introducing, using and embedding intervention components into clinical practice.

The mixed‐methods evaluation consisted of audit, patient health record review, interviews and focus groups. The audit found 35% of parents were aware of C4H, an increase from the pre‐implementation period when two audits showed 19% and 6% awareness.^14^ This result represented the success achieved within the first 6 months. Although the goal set of 50% awareness was not achieved, subsequent monthly auditing has shown parent awareness to now be greater than 85%. Overall, this represents a positive effect of implementation and compares favourably to the level of family awareness following implementation of similar programmes.^5^, ^6^

The patient health record review confirmed the C4H process was used. The more formal verbalization of parents' concern may have also influenced staff decision to escalate care and place calls for MET reviews, although from this retrospective review it was not possible to confirm or refute this. There was a small increase in the documented involvement of parents in escalation of care. The C4H process as now implemented also appeared feasible and acceptable with positive views expressed by parents who had been involved when their children had suffered clinical deterioration. Nurses were also positive about the C4H process having synergy with their family‐centred care practises and previously held reservations about over or misuse of C4H had decreased.

Remaining C4H barriers were related to beliefs and to process. Interviewed parents identified that even though they knew how to use C4H, they found it difficult to challenge or speak assertively to health professionals. Norms of passivity, language and health literacy have been described to influence patient and family's willingness and ability to actively speak up^29^ in similar situations. It has also been suggested that families' reticence to speak up is associated with confidence and familiarity with the health‐care system.^12^, ^15^ In this study, difficulty speaking up was also described by parents who were themselves highly health literate health professionals who wanted to avoid conflict with colleagues. At this point, no specific strategies had been used to address the barrier of overcoming the traditional hospital hierarchy which remains a widely reported issue in escalation of care.^30^, ^31^, ^32^, ^33^, ^34^

There were some unexpected findings related to nurse beliefs and behaviours. Despite nurses knowing that there had been only one C4H direct parent MET call since implementation, this concern remained a factor that influenced their decisions to inform parents. Nurses selected to inform only parents who they considered would benefit most from the C4H information. It appears that this belief remained a barrier and highlights the challenge of changing beliefs. A further unanticipated and concerning consequence of implementing C4H was the use of the C4H pathway by nurses. Many reports have highlighted that communication problems between health professionals themselves present barriers to effective escalation of care.^30^, ^31^, ^33^, ^35^ The described practice by nurses encouraging parents to use C4H to obtain a medical review or even utilizing C4H themselves seems reflective of such interprofessional communication barriers. These findings urgently require further exploration.

For C4H to be used as intended, in addition to the multiple strategies to inform parents about the process, the next steps will be to address the more challenging issues of supporting and enabling parents to be able to speak up when concerned and target nurse behaviours and interprofessional communication barriers. Programmes advocating open communication between consumers and health professionals have been recommended.^12^ In the paediatric setting, this involves full collaboration between parents and health professionals that had not been achieved in the study setting. Further work is required to fully integrate the C4H process with health professionals' responses. Suggested strategies include promoting nurse engagement with parents as a routine component of clinical assessment as well as development and implementation of contextually appropriate tools to support parents to confidently participate in their child's assessment and recognition of early signs of deterioration. If the interprofessional communication barriers themselves are not addressed, promoting parent involvement in the context of escalation of care for clinical deterioration may be destined to failure.

Limitations of this pragmatic study include that it was undertaken after the C4H process had been already introduced. This necessitated a responsive approach for both implementation and evaluation. The dynamic nature of the health‐care context created challenges to fully understanding the implementation and which components were most effective to result in the observed changes. It was not possible to control fidelity or dose of all the implementation strategies used. In order to deal with these contextual challenges, a widely used theory‐informed health‐care improvement methodology was used. Collection of data using retrospective record review may have missed a greater parental involvement than recorded. Convenience sampling of parents prevented data saturation being confirmed. Meaningful engagement of the entire health‐care team was not achieved. Greater benefit over time may still be realized in terms of parent awareness of C4H and capacity and confidence to speak up when concerned about clinical deterioration as experience and exposure to components of the implementation strategy take hold.

## 5 CONCLUSION

This study demonstrated the application of health‐care improvement principles to post hoc implementation of the C4H process. Irrespective of whether parents actively used it or not, there was a reasonable and improving level of parent awareness of the C4H process, which was viewed positively by parents and nurses alike. To achieve a higher level of parent awareness in a sustainable way, multiple strategies are required. Although parents preferred to be informed by nurses at the time of admission to the ward, nurses considered this to be unrealistic to achieve and it was not routinely undertaken. Nurses selectively chose who to inform and who not to inform based on their judgement of parents who would benefit from the information.

The study highlighted that a much more difficult undertaking is for parents to feel confident enough to actually use C4H to speak up when concerned about their child's clinical deterioration. This is yet to be realized. Further work is required to understand how to achieve this, especially for more vulnerable parents. Importantly, strategies are urgently needed to address the interprofessional communication problems that continue to delay effective escalation of care. Involving parents in recognition and response to clinical deterioration should add an additional safety net to the care of sick children.

## Data Availability

This is a revision and submitted prior to this policy.
