# Supplementary material for: Parent escalation of care for the deteriorating child in hospital: A health‐care improvement study
Source: Health Expect. 2019 Jul 16;22(5):1078–88. doi: 10.1111/hex.12938 (PMC6803393; doi:10.1111/hex.12938)
Supplement: Supplementary file 1 [file HEX-22-1078-s001.docx]

| **Parent**   - Were you aware of C4H? - How did you find out about C4H? - What was your experience in hospital when your child was deteriorating/getting sicker? - What is the best way for parents to find out about C4H? - Should we do anything differently? - Were you satisfied with the experience/ care you received?   **Nurses**   - Are you aware of C4H? - Do you inform parents? - Do you give out the parent brochure? - When do you inform parents / give out the brochure? If not why not? - Have you been involved with parents who have used C4H? - How do you think parents should be informed of C4H? - How can we increase parent awareness? - What are the benefits and negatives to C4H? - Do we need to do anything differently? |
| --- |

Appendix Interview guide
